# scSemiPLC: a semi-supervised learning framework for annotating single-cell RNA-Seq data by generating pseudo-labels through clustering

**DOI:** 10.1128/msystems.00223-25

**Published:** 2025-12-08

**Authors:** QianYi Ma, LinJie Wang, Wei Li

**Affiliations:** 1 School of Computer Science and Engineering, Northeastern University 12434, Shenyang, China; 2 Key Laboratory of Intelligent Computing in Medical Image (MIIC), Northeastern University 12434, Shenyang, China; NYU Langone Health, New York, New York, USA

**Keywords:** scRNA-seq data, semi-supervised learning, cell annotation, pseudo-label

## Abstract

Single-cell RNA sequencing (scRNA-seq) technology enables researchers to explore heterogeneity of diverse cell types within complex tissues at the single-cell resolution. Cell annotation, as a crucial step in scRNA-seq data analysis, provides biologically meaningful cell identity information for biological research. With the proliferation of publicly available datasets and the expansion of sequencing data scale, traditional annotation methods reliant on manual marker gene matching have become increasingly cumbersome and time-consuming. Consequently, efficient and convenient automated cell annotation methods are gradually becoming mainstream. In this paper, we propose a single-cell semi-supervised annotation training framework called scSemiPLC, which generates pseudo-labels through clustering and consistency regularization. Specifically, scSemiPLC utilizes existing label information to guide the clustering of unlabeled data. During model training, it assigns pseudo-labels to the unlabeled samples and constrains the prediction of perturbed data to be similar to the pseudo-labels. This strategy addresses the low utilization of unlabeled data caused by the fixed high threshold pseudo-labeling paradigm, offering a new approach for cell annotation in the semi-supervised learning field. Experimental results demonstrate the superior performance of scSemiPLC in annotation accuracy and stability, extraction of biologically meaningful representations, and robustness to the number of cell labels, significantly outperforming classical automatic annotation and mainstream semi-supervised learning methods.

**IMPORTANCE:**

This work proposes a novel cell annotation training framework, scSemiPLC, which significantly enhances the efficiency and accuracy of annotation by fully leveraging unlabeled data. In the semi-supervised learning component, the framework innovatively generates pseudo-labels through clustering. Subsequently, it evaluates the reliability of these pseudo-labels and assigns corresponding weights, thereby balancing both their quantity and quality. This approach provides new insights into the direction of automatic cell annotation within the realm of semi-supervised learning.

## INTRODUCTION

Single-cell RNA sequencing (scRNA-seq) technology has experienced significant and rapid development in recent years, and its applications in various scenarios within the biomedical field have become increasingly widespread (1, 2). By analyzing the variations within the transcriptome across cells, this technology can measure gene expression at the single-cell level, detect the heterogeneity of different cell types, and reveal the population structures and cell dynamics hidden within complex tissues (3, 4). An essential step in scRNA-seq data analysis is cell annotation. Cell type identification is a prerequisite to studying heterogeneous cell populations, also serving as the basis for valuable biological discoveries related to gene function and disease mechanisms (5).

The traditional cell annotation method is divided into three steps. First, unsupervised clustering is employed to obtain multiple cell clusters. Then, differential expression analysis is performed among the cell clusters to identify cluster-specific marker genes. Finally, cells are manually annotated according to the ontology information associated with the identified marker genes from the latest databases. Although this process can identify cell types, manual annotation still has some limitations. (i) Unsupervised clustering methods are data-driven and fail to incorporate biological significance. This makes it challenging to find the best match between the marker genes of each cluster and the typical markers of specific cell types, resulting in clustering results not accurately corresponding to cell types (6, 7). (ii) Different subtypes of cells are highly similar in gene expression, which makes it difficult to distinguish them relying on individual subjective decisions accurately. (iii) The annotation process requires manual inspection and verification against annotation databases, which is labor-intensive and necessitates background knowledge of cell type-specific markers. To address these challenges, some automated cell annotation tools and methods have emerged as alternative solutions.

Currently, automatic cell annotation methods can be broadly categorized into two types. An approach is marker-based annotation, which discovers various possible cell types in data to be annotated using known marker genes expressed explicitly by cells. The known associations between marker genes and cell types can be obtained from databases, such as CellMarker (8) and singleCellBase (9). These methods involve probabilistic allocation to identify cell types, enabling prior knowledge transfer when reliable cell type markers are available. For example, SCINA (10) operates at the cluster level by fitting bimodal distributions of marker genes, and CellAssign (11) uses Bayesian probabilistic models at the single-cell level. Another approach is reference-based annotation, which compares the unannotated scRNA-seq data (the query data) with expert-annotated scRNA-seq data (reference data). These approaches transfer labels from the reference cells (clusters) to sufficiently similar query cells (clusters) based on their correlations. Current reference-based annotation tools mainly perform single-cell and cluster referencing. The former associates each cell in the query data with annotated clusters in the reference data, such as scmap (12) and SingleR (13), while the latter cross-links unannotated clusters with annotated reference clusters, such as CIPR (14). Besides data comparison, cell type labels can be assigned to samples in the query data by employing machine learning tools trained via supervised learning on reference data. For example, ACTINN (7) employs a neural network that is trained on a reference dataset and predicts cell types for the query dataset based on trained parameters. Other commonly used algorithms include random forests (15), K-nearest neighbors (16), support vector machines (17), and Transformers (18) for large-scale datasets. In practice, both of these automatic cell annotation methods impose stringent requirements on the specific expression of marker genes and the quality of annotations within the reference data. However, obtaining a large amount of well-annotated data that meets these criteria is a challenge. To fully leverage existing cell labels and the large amount of unlabeled data for better data interpretation, researchers are increasingly preferring semi-supervised learning methods. scSemiCluster (19) is a semi-supervised annotation training framework consisting of deep generative, discriminative clustering, and pairwise constraints. The clustering solution of the query data is constrained by the structural regularization of the reference data to utilize labeled data effectively. CALLR (20) combines the graph Laplacian matrix with sparse logistic regression for semi-supervised annotation of scRNA-seq data. The adversarial training has also been applied to annotate cell types. scSemiGAN (5) applies adversarial training to cell annotation and introduces a supervised label prediction loss to guide the training of the generative adversarial network. All the aforementioned automatic cell annotation methods are guided by labeled data and fully incorporate unlabeled data into model training through diverse strategies, thus enhancing model performance. Here, we focus on the generation of pseudo-labels for unlabeled data (21), a classic semi-supervised learning strategy that represents an unattempted perspective in semi-supervised cell annotation.

In addition to using a small amount of labeled data, the pseudo-label strategy selects high-confidence samples from the unlabeled data based on specific criteria. The predictions for these samples are treated as artificial labels, thereby participating in model training in a supervised way. The model generates a significant number of incorrect annotations when producing pseudo-labels. Many algorithms, such as UDA (22) and FixMatch (23), use fixed high thresholds to filter high-confidence pseudo-labels. While this strategy effectively handles label noise issues by ensuring high-quality pseudo-labels participate in model training, it results in the underutilization of unlabeled data. This occurs because only a few of the unlabeled samples exceed the confidence threshold during training. Furthermore, the inconsistency between the number and learning difficulty across different categories within a dataset suggests that categories with a large proportion or easier learning within a batch receive more high-confidence pseudo-labels. This leads to bias during the model training process, resulting in significant differences in prediction performance among different categories or even model collapse. Therefore, it is crucial to generate enough pseudo-labels covering diverse classes in the training process. The quantity and quality of the pseudo-labels produced by the model are the key factors affecting the performance of semi-supervised learning algorithms.

To address the above issues, we propose a new single-cell semi-supervised annotation training framework, scSemiPLC. This framework adopts a novel cluster-based (24) pseudo-labels generation strategy and evaluates the confidence of pseudo-labels, significantly improving the utilization of unlabeled data. Moreover, we pretrain the model using contrastive learning, maximizing mutual information between different data views to learn robust data representations and improve training efficiency. Experiments comparing scSemiPLC against existing cell annotation methods, including supervised and semi-supervised learning approaches, across several public datasets demonstrate that scSemiPLC achieves higher accuracy with limited annotated data, confirming its superior performance.

## MATERIALS AND METHODS

### Datasets and data preprocessing

In this paper, we use eight publicly available scRNA-seq datasets. To study the generalization capability of the methods, we collect datasets covering different tissue types from multiple organisms sequenced using various protocols. The detailed information of the scRNA-seq datasets is shown in Table 1. Specifically, we select three mouse scRNA-seq datasets from Tabula Muris (25), covering bladder, kidney, and tongue tissues. The Baron Mouse and Human datasets represent pancreatic tissue derived from the inDrop protocol (26). Chen et al. sequenced the mouse hypothalamus by the Drop-seq protocol, yielding a large dataset comprising over 14,000 cells across 15 distinct cell types (27). For human peripheral blood mononuclear cells (PBMCs), we collected two different datasets from 10X Genomics (28) and SeqWell (29) protocols, respectively. The PBMC_10X butler2018pbmc10x dataset is downloaded from the 10X Genomics website and preprocessed by the built-in functions in the toolkit scanpy (30). All datasets contain manually annotated cell types, which serve as the gold standard for evaluating the cell annotation performance of methods.

**TABLE 1:** Summary of the dataset after preprocessing

Dataset	Organ/tissue	Protocol	Cell × Gene	Cell types
Baron Mouse (26)	Pancreatic islets	InDrop	1,886 × 2,831	13
Bladder (25)	Bladder	10x Genomics	2,500 × 2,183	4
PBMC_10x (28)	Blood	10x Genomics	2,638 × 1,838	9
Kidney (25)	Kidney	10x Genomics	2,781 × 1,988	8
PBMC_SeqWell (29)	Blood	SeqWell	3,694 × 6,713	6
Tongue (25)	Tongue	10x Genomics	7,538 × 1,117	3
Baron Human (26)	Pancreatic islets	InDrop	8,569 × 1,864	14
Chen (27)	Hypothalamus	Drop-seq	14,419 × 2,774	15

Data preprocessing involves three main steps, including filtering, standardizing, and selecting highly variable genes. First, cells with fewer than 200 expressed genes and genes expressed in fewer than three cells are excluded. Second, the gene expression data of all cells are standardized to make them comparable among different cells. Then, genes are sorted given their standard deviation, and those with high expression variability are selected as the final genes. Finally, the data are scaled by unit variance and zero mean. The labeled and unlabeled data are defined as follows:


(1) $$S=\left\{\left(X^{s},\;Y^{s}\right)\right\},\;T=\left\{X^{t}\right\}$$

where $X^{s}\in {\mathbb{R}}^{N_{s}\times D}$,$X^{t}\in {\mathbb{R}}^{N_{t}\times D}$ are the gene expression metrics, serving as input to the model. $N_{s}$ and $N_{t}$ are the number of cells in S and T, respectively, D is the number of genes. $Y^{s}\in {\left\{1,2,\cdots \;,C\right\}}^{N_{s}}$ is the annotations within labeled data, where C is the number of categories. In addition, ${\hat{Y}}^{t}$ signifies the prediction result of the model on unlabeled data T during the training stage.

### General framework

**Fig 1:**
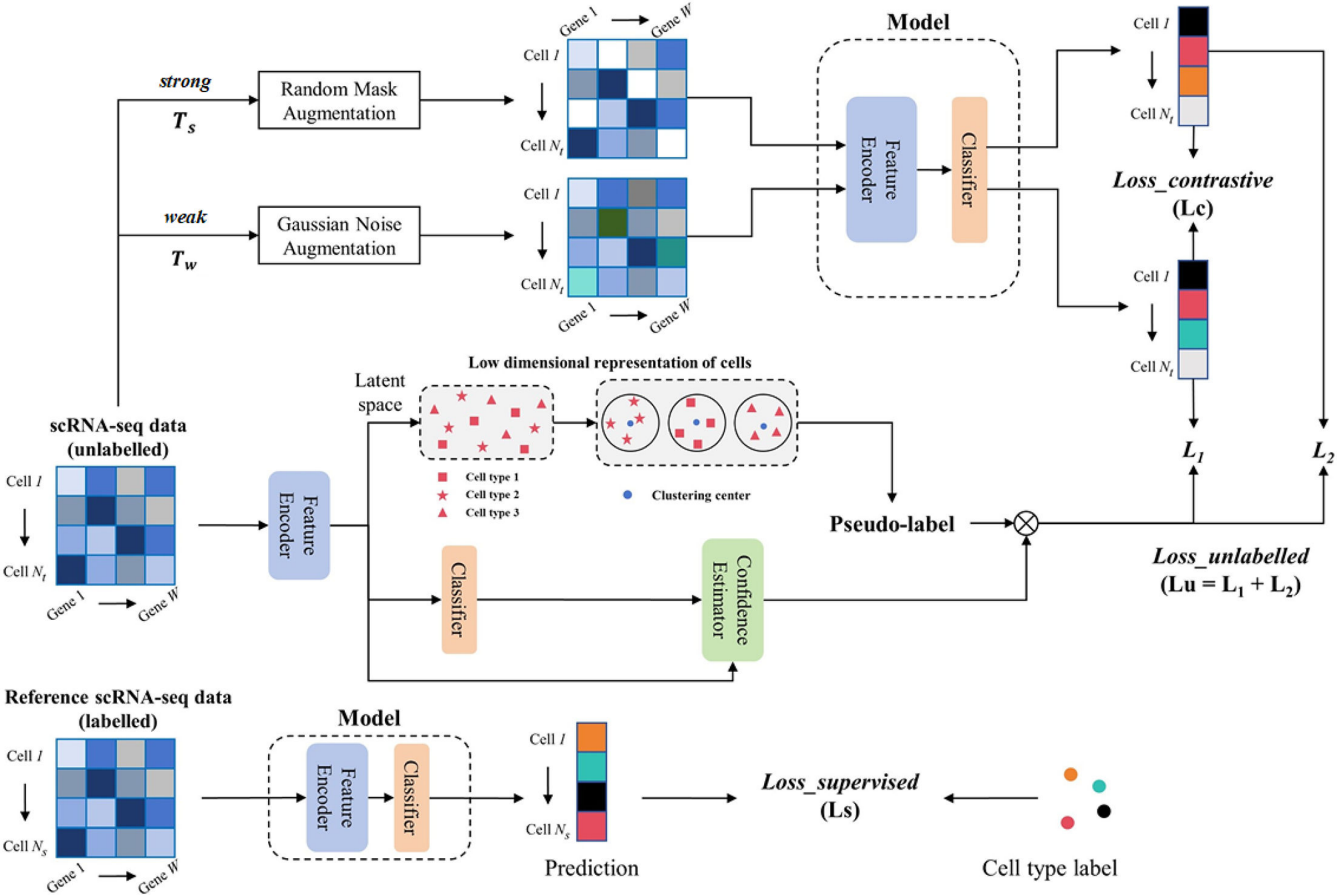
The scSemiPLC framework utilizes both labeled and unlabeled data for training. Unlabeled data undergo augmentation through random masking and Gaussian noise addition, and pretraining is conducted via contrastive learning between model prediction outputs. In addition, the embeddings of the unlabeled data are extracted and clustered, and the pseudo-labels produced by the clusters are used to guide the further training of the model predictions. For labeled data, training is performed by supervised learning.

Considering cell annotation as a classification task, the proposed training framework scSemiPLC employs the ACTINN (7) network, a four-layer multi-layer perceptron (MLP), as the basic model Φ for feature extraction and cell type recognition. $\phi_{l}\left(X\right)$ denotes the output of vector X at the l-th layer within this model. As shown in Fig. 1, the training framework consists of three parts, which are elaborated in the following section.

#### Pretraining based on contrastive learning

To achieve higher annotation performance with minimal labeled data, pretraining the model can be accomplished using unlabeled data. Contrastive learning distinguishes data in an abstract semantic space, demonstrating impressive results across various research domains. The first part of scSemiPLC is the unsupervised pretraining based on contrastive learning, where batches are formed through random sampling from unlabeled data. Two distinct data augmentations are performed on N samples in a batch to obtain N pairs of samples, i.e., a total of 2N samples. The core idea of contrastive learning is to ensure that the distances between the two augmentations from the same sample are close, while maintaining a considerable distance from the other 2(N−1) augmentations in the batch.

As presented in Fig. 1, the contrastive learning-based pretraining framework consists of three components: data augmentation T(·), annotation network Φ (with network parameters $\theta_{\phi }$), and contrastive learning loss $L_{c}$. In terms of data augmentation, unlike SimCLR (31), which uses the same perturbation to obtain two different augmented results, scSemiPLC employs a weak augmentation $T_{w}(\cdot )$ and a strong augmentation $T_{s}(\cdot )$ with differing degrees of perturbation. Specifically, $T_{w}(\cdot )$ is implemented by adding Gaussian noise, which introduces a mild perturbation while preserving the majority of the original information. It introduces random values from a normal distribution with a mean of 0 and a standard deviation of 0.5 to half of the gene expression vectors of cells, with a probability of 0.8. $T_{s}(\cdot )$ employs random masking to drop some gene expression values by setting them to zero at the same probability and proportion (32, 33). With this strategy, weak augmentation provides stable data information. In contrast, strong augmentation enforces the model to overcome heavy perturbations to fit this information, ensuring the robustness of the learned representation (23).

The augmented data $T_{w}(x_{i})$ and $T_{s}(x_{i})$ are fed into Φ separately to obtain embedding $z_{i}$ and $z_{j}$ for contrastive learning loss calculation, which is defined as:


(2) $$L_{c}=-\log\frac{exp\left(sim\left(z_{i},z_{j}\right)/\tau \right)}{\sum_{k=1}^{2N}1_{\left(k\neq i\right)}exp\left(sim\left(z_{i},z_{k}\right)/\tau \right)},$$

where sim(⋅) is a similarity measure function, τ is an adjustable temperature parameter used to scale the range of similarity, and $1_{\left(k\neq i\right)}$ is an indicator function that takes the value of 1 if k≠i and 0 otherwise.

#### Production of pseudo-label based on clustering

For the clustering strategy, we first calculate the cluster centers $\{O_{c}^{s}{\}}_{c=1}^{C}$ for different cell types using the embeddings of the labeled data and assign them as the initial cluster centers $\{O_{c}^{t}{\}}_{c=1}^{C}$ for the unlabeled data. The unlabeled data $X^{t}$ is input into the model, and the output $\phi_{2}\left(X^{t}\right)$ from the second layer of model Φ is extracted as input for the spherical K-means algorithm to cluster the unlabeled samples. This process iteratively performs label assignment and cluster center updates.

#### Algorithm 1: spherical K-means algorithm

**Input:** labeled data $X^{s}$, unlabeled data $X^{t}$

**Output:** Cluster centers ${\left\{O_{c}^{t}\right\}}_{c=1}^{C}$

1: $O_{c}^{s}=\sum_{i=1}^{N_{s}}1_{Y_{t}^{s}=c}\frac{\phi_{2}(x_{i}^{s})}{||\phi_{2}(x_{i}^{s})||}$

2: $O_{c}^{t}\leftarrow O_{c}^{s}$

3: **while <** maximum iterations and unconvergence **do**

4: ${\hat{y}}_{i}^{t}\leftarrow {arg\;min}_{c}\;dist(\phi_{2}(x_{i}^{t}),O_{c}^{t})$

5: $O_{c}^{t}\leftarrow \sum_{i=1}^{N_{t}}1_{{\hat{y}}_{i}^{t}=c}\frac{\phi_{2}(x_{i}^{t})}{||\phi_{2}(X_{i}^{t})||}$

6: **end while**

7: **return** Cluster centers ${\left\{O_{c}^{t}\right\}}_{c=1}^{C}$

Each cell within the unlabeled data is assigned a label until convergence, or the maximum iterations are reached. The distance between cells in the feature space is measured by cosine similarity:


(3) $$dist\;(a,b)=\frac{1}{2}\left(1-\frac{\left\langle a,b\right\rangle }{||a||\;||b||}\right)$$

Detailed steps of clustering are shown in Algorithm 1. After obtaining the cluster labels, we further introduce a learnable method to measure pseudo-label confidence and define an additional network (34) to learn confidence estimation:


(4) $$e\left(X^{t}\right)=e\left(\phi_{2}\left(X^{t}\right),\Phi \left(X^{t}\right);\theta_{e}\right)$$

where e(·) is the confidence estimator with model parameters $\theta_{e}$. It accepts $\phi_{2}\left(X^{t}\right)$ and $\Phi \left(X^{t}\right)$ as the input features and logits, as illustrated in the Fig. S1.

#### Consistency regularization based on data augmentation

For each unlabeled sample, consistency regularization requires that the output before and after randomly injected noise approximate each other. This training strategy can improve the generalization ability and enhance the robustness of the model. $T_{s}\left(\cdot \right)$ and $T_{w}\left(\cdot \right)$ two data augmentations are employed in scSemiPLC. The cross-entropy loss function trains the model to match the predictions $\Phi \left(T_{w}\left(X^{t}\right)\right)$ and $\Phi \left(T_{s}\left(X^{t}\right)\right)$ with the pseudo-labels ${\hat{Y}}^{t}$ obtained from clustering. The confidence of these pseudo-labels is incorporated into training by a weighted loss function:


(5) $$\begin{array}{rl} L_{u}= & e\left(X^{t}\right)\cdot H\left({\hat{Y}}^{t},\Phi \left(T_{w}\left(X^{t}\right)\right)\right)+\\ & e\left(X^{t}\right)\cdot H\left({\hat{Y}}^{t},\Phi \left(T_{s}\left(X^{t}\right)\right)\right) \end{array}$$

where H(⋅,⋅) is the cross-entropy between two probability distributions.

### Framework training strategy

Upon the construction of the entire framework, we provide the training strategy of the scSemiPLC and the training objectives at different stages. Besides $L_{c}$ and $L_{u}$, the supervised learning loss for labeled data is defined as $L_{s}=\frac{1}{N}\sum_{i=1}^{N}H\left(y_{i}^{s},\Phi \left(x_{i}^{s}\right)\right)$. The scSemiPLC training process comprises the following three stages.

Stage 1, train the annotation model Φ using unlabeled data through contrastive learning, with optimal parameter ${\hat{\theta }}_{\phi }$ derived from the formula:


(6) $$\left({\hat{\theta }}_{\phi }\right)={argmin}_{\theta_{\phi }}L_{c}.$$

Stage 2, train the annotation model Φ using labeled data through supervised learning, with optimal parameter ${\hat{\theta }}_{\phi }$ derived from the formula:


(7) $$\left({\hat{\theta }}_{\phi }\right)={argmin}_{\theta_{\phi }}L_{s}.$$

Stage 3, after a certain number of epochs of supervised learning, the confidence estimator e is trained using unsupervised data through consistent regularization. Then, the annotation model Φ is trained using the total data through supervised learning and consistent regularization. It is important to note that during the training of the confidence network e, the annotation model Φ is frozen, and conversely, when training the annotation model Φ, the confidence network e is frozen. This alternating optimization strategy ensures the stability of scSemiPLC training. The optimal parameters ${\hat{\theta }}_{\phi }$ and ${\hat{\theta }}_{e}$ can be formulated by:


(8) $$\left({\hat{\theta }}_{\phi }\right)={argmin}_{\theta_{c}}\left(L_{s}+\lambda L_{u}\right)$$

(9) $$\left({\hat{\theta }}_{e}\right)={argmin}_{\theta_{e}}L_{u}.$$

## RESULTS AND DISCUSSION

### Competing methods and evaluation index

Taking scSemiPLC as a semi-supervised learning method, we compare it with three semi-supervised learning methods, such as scSemiCluster (19), CALLR (20), and scSemiGAN (5). Additionally, we include two reference-based methods, including scmap (12) and SingleR (13), and a supervised learning-based method, SingleCellNet (15), for comparison. Cell annotation, as a multi-class classification issue, is evaluated by accuracy and F1-score, with higher values indicating superior annotation performance. The F1-score is robust to sample imbalance, thus enabling reliable verification of the model’s comprehensive annotation capacity.

We conduct experiments on the same machine equipped with an NVIDIA GeForce RTX 4090D GPU to ensure fairness. All comparison methods are trained strictly following the parameters provided in their respective papers or code and receive preprocessed data consistent with scSemiPLC. In scSemiPLC, the three stages of the training process run for 100, 200, and 150 epochs, respectively. The learning rates of the Adam optimizer for model Φ are 5e-4, 1e-4, and 5e-5 in these three stages, while the learning rate for the estimator e is 1e-4. The parameter λ in the loss function defaults to 0.5.

### Comparison with other annotation methods

For each real dataset, we employ stratified cross-validation, randomly grouping the dataset based on cell type proportions, with each group comprising 10% of the dataset. Sequentially, we select a group of cells as labeled data and use remaining cells as unlabeled data. Table 2 and Table S1 present the annotation accuracy and F1-score of different methods on eight real datasets.

**TABLE 2:** Performance of cell annotation methods across datasets^*a*^

Type	Method	Baron Mouse	Bladder	PBMC_10x	Kidney	PBMC SeqWell	Tongue	Baron Human	Chen
Ref-based	scmap-cluster	82.672 ±0.531 ∗∗	98.464 ±0.130 ∗∗	79.629 ±0.574 ∗∗	94.308 ±0.230 ∗∗	30.623 ±0.864 ∗∗	94.586 ±0.120 ∗∗	89.224 ±0.362 ∗∗	76.213 ±0.266 ∗∗
	scmap-cell	92.322 ±0.252 ∗∗	98.124 ±0.231 ∗∗	75.478 ±0.435 ∗∗	95.361 ±0.133 ∗∗	74.553 ±0.722 ∗∗	97.174 ±0.065 ∗∗	95.730 ±0.050 ∗∗	83.103 ±0.221 ∗∗
	SingleR	85.376 ±0.899 ∗∗	99.680 ±0.030 ∗∗	75.504 ±0.676 ∗∗	96.764 ±0.236 ∗∗	83.311 ±0.303 ∗∗	90.608 ±0.652 ∗∗	92.366 ±0.259 ∗∗	82.703 ±0.154 ∗∗
Supervised	SingleCellNet	88.420 ±0.622 ∗∗	97.688 ±0.151 ∗∗	80.144 ±0.440 ∗∗	94.671 ±0.420 ∗∗	89.169 ±0.073 ∗∗	87.373 ±0.510 ∗∗	92.348 ±0.207 ∗∗	84.245 ±0.126 ∗∗
Semisupervised	scSemiCluster	95.371 ±0.259 ∗∗	99.740 ±0.045 ∗∗	77.142 ±0.656 ∗∗	98.519 ±0.109 ∗∗	OCMM	97.858 ±0.098 ∗∗	97.643 ±0.124 ∗∗	88.451 ±0.244 ∗∗
	CALLR	92.137 ±0.381 ∗∗	98.752 ±0.100 ∗∗	71.941 ±2.416 ∗∗	98.511 ±0.150 ∗	90.003 ±0.170 ∗∗	97.802 ±0.056 ∗∗	93.311 ±0.211 ∗∗	86.474 ±1.615 ∗
	scSemiGAN	92.322 ±0.554 ∗∗	99.544 ±0.120 ∗∗	73.169 ±0.955 ∗∗	98.533 ±0.187 ∗	88.362 ±0.413 ∗∗	98.110 ±0.071 ∗∗	97.620 ±0.063 ∗∗	89.577 ± 0.136
	scSemiPLC	**96.909 ±0.160**	**99.900 ±0.012**	**83.548 ±0.282**	**98.975 ±0.151**	**91.061 ±0.294**	**98.346 ±0.055**	**98.363 ±0.045**	**89.893 ±0.118**

^*a*^ The data in the table show the average accuracy (standard error) of the 10-fold cross-validation. Bold text marks the best models on each dataset. OCMM indicates that the original count matrix required by scSemiCluster is missing from the PBMC_SeqWell dataset. The t-test is conducted to compare the performance of each method against scSemiPLC. Statistical significance is indicated with ** for *P* < 0.01 and * for *P* < 0.05.

We compare scSemiPLC with three other semi-supervised learning methods. As shown in Table 2 and the Table S1 scSemiPLC achieves the best annotation accuracy in all scRNA-seq datasets, outperforming other methods. In the PBMC_10x and Baron Mouse datasets, there is a significant difference in F1-score among the four semi-supervised learning-based methods, with differences of 23.1% and 24.9% between the highest and lowest scores, respectively. However, in the Bladder and Tongue datasets, the maximum difference in accuracy and F1-score among these four methods is only 1.2% and 3.1%. The number of cell types in the first two datasets is 9 and 13, respectively. In comparison, in the latter two datasets, the number of cell types did not exceed 4. scSemiPLC achieves optimal results regardless of the number of cell types and performs exceptionally well on the PBMC_10x, Baron Mouse, and Baron Human datasets with more cell types, where the F1-score surpasses the second-best method by 13%, 11%, and 5%, respectively. Thus, scSemiPLC demonstrates strong robustness when handling datasets with different numbers of cell types, particularly when facing datasets rich in cell types. The better annotation performance benefits from the production strategy of pseudo-labels. scSemiPLC calculates the initial clustering centers for each cell type within the labeled data, so that the annotation effects of various cell types are adequately considered during the training process.

**Fig 2:**
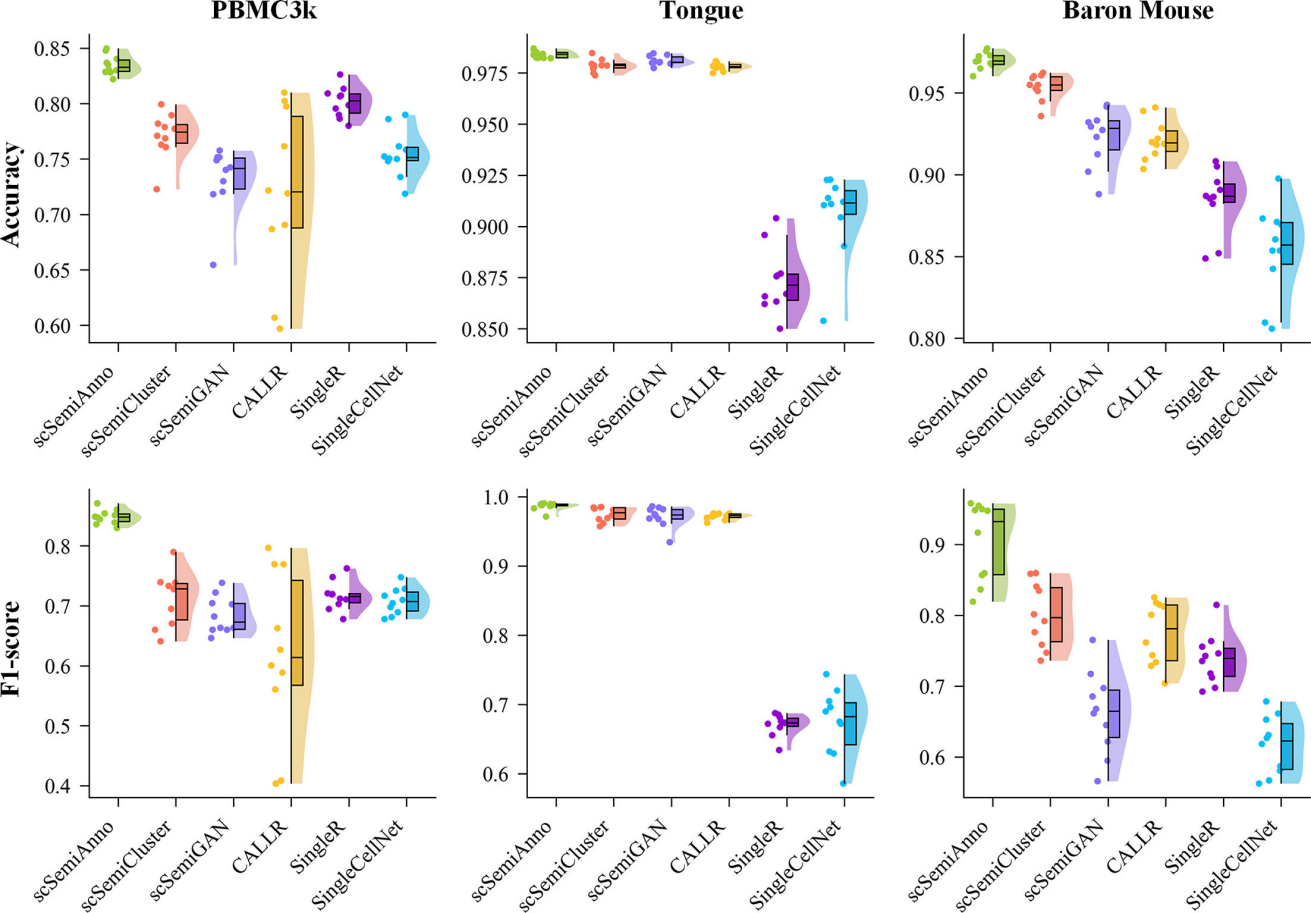
Raincloud plots of cell annotation performance from different methods on the PBMC_10x, Tongue, and Baron Mouse datasets.

We further compare scSemiPLC with reference-based annotation methods. Since scmap-cell and scmap-cluster could only annotate a portion of the cells, we evaluated these methods by considering ”unassigned” as the predicted label for unannotated cells. Although the scores are lower, the number of predicted cells is unified, which also indicates that the method with a fixed threshold of similarity has certain drawbacks. Overall, the annotation performance of these methods is less satisfactory compared with the aforementioned annotation methods based on semi-supervised learning, and they only performed well in individual datasets. For example, scmap-cluster achieves the second-highest F1-score in PBMC_10x, SingleR performs well in Bladder, and SingleCellNet achieves the second-highest accuracy in PBMC_10x among all compared methods, but their performance is not consistently high. To provide a more intuitive view of the data distribution of different method predictions, we present raincloud plots of four semi-supervised methods, reference-based SingleR, and the supervised learning-based SingleCellNet on the PBMC_10x, Tongue, and Baron Mouse datasets. The results in Fig. 2 demonstrate that scSemiPLC exhibits excellent and robust performance compared to other methods. Furthermore, t-test comparisons with other methods confirm that most superiority of scSemiPLC is statistically significant.

### Latent space features analysis

**Fig 3:**
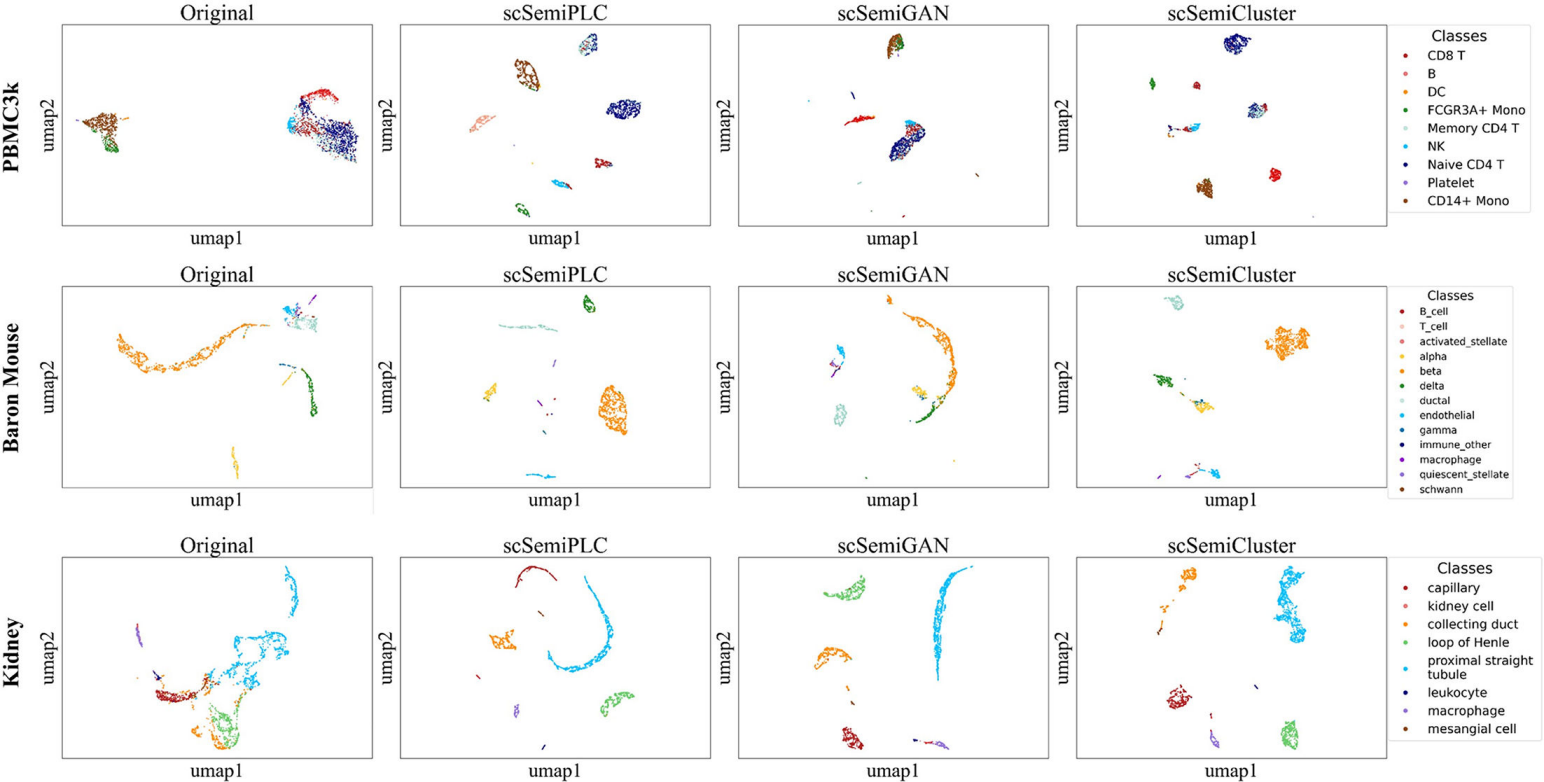
UMAP visualizations of original data, scSemiPLC, scSemiGAN, and scSemiCluster on the PBMC_10x, Baron Mouse, and Kidney datasets colored by cell type labels.

Along with three other semi-supervised learning-based methods, scSemiPLC is built on neural networks, all of which include a feature extraction module in the model. To explore the specificity of representation extracted by different methods in the embedding space and assess whether they have sufficient discriminability to aid in cell type identification, we use UMAP (35) to visualize these representations with ground truth labels (Fig. 3). We compare the representations extracted by scSemiPLC, scSemiGAN, and scSemiCluster, as well as the original data, on Baron Mouse, PBMC_10x, and Kidney datasets.

The subsets of T cells in the PBMC_10x dataset, including CD8 T, memory CD4 T, and naive CD4 T cells, belong to similar cell types, which makes annotation more challenging, as demonstrated in Fig. 3. The embeddings generated by scSemiGAN for these three cell types are mixed, making them almost indistinguishable. The scSemiCluster partially separates Naive CD4 T (dark blue) and CD8 T (dark red) cells, but some of these two types of cells are still mixed with Memory CD4 T cells. Additionally, the clustering effect for CD8 T cells is also inferior, with most samples dispersed among three clusters rather than forming a single cluster. In contrast, the separation of these three cell subtypes of T cells in the latent space generated by scSemiPLC achieves the best performance, with each subtype almost forming a distinct cluster. Similarly, only scSemiPLC achieves individual clustering of NK cells with minimal mixing. These results indicate that through contrastive learning pretraining and tuned learning of clustering pseudo-labels, scSemiPLC can better distinguish cells of similar types, thereby improving the annotation performance.

In the representation of the original data in the Baron Mouse dataset, we observe that the alpha (yellow), beta (orange), gamma (blue), and delta (green) cell types are generally clustered separately. This characteristic is preserved and further strengthened by scSemiPLC. However, these four cell types are mixed and difficult to distinguish in the representation generated by scSemiGAN. Although scSemiCluster can separate most beta and delta cells into single clusters, alpha and gamma cells are mixed, resulting in lower accuracy compared with scSemiPLC. For cell types that are indistinguishable in the original representation, such as quiescent_stellate, endothelial, and immune_other cells, scSemiPLC can separate them well, whereas scSemiGAN and scSemiCluster do not perform as effectively. These results indicate that scSemiPLC can learn a biologically meaningful representation. For the Kidney dataset, the embedding representations of the three methods generally achieve clustering of each category separately. However, scSemiGAN and scSemiCluster exhibit different degrees of confusion when separating kidney cells (pink) and macrophages (purple), as well as kidney collecting duct epithelial cells (yellow) and mesangial cells (brown). The visualization results in the latent space by scSemiPLC are most satisfactory, supporting the highest annotation accuracy and F1-score of cell annotation.

**Fig 4:**
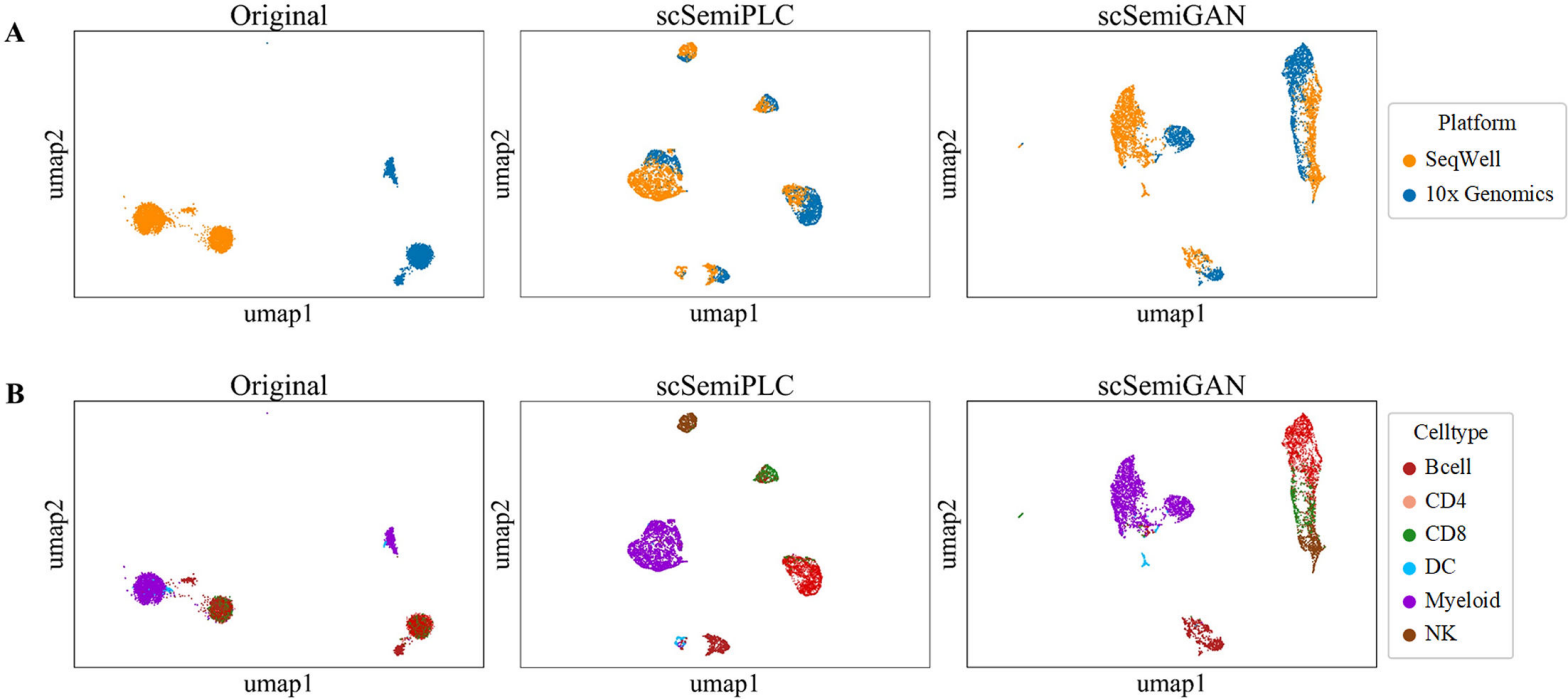
UMAP visualizations of original data, scSemiPLC and scSemiGAN on the integrated PBMC data, comprising PBMC_10x and PBMC_SeqWell datasets, colored by (**A**) technical platform and (**B**) cell type labels.

The scRNA-seq data are typically compiled from multiple experiments, with differences in capture time, personnel handling, reagent batches, equipment, and even technical platforms. These differences result in significant data variability or batch effects, which can confound valuable biological changes during data integration (36). To investigate the robustness of scSemiPLC against the irrelevant factors, we select two human PBMC datasets, PBMC_10x and PBMC_SeqWell, captured by the 10X and SeqWell platforms, respectively. As shown in Fig. 4, in the original latent space, there are significant differences in the data distribution between the two technical platforms, without cells mixed. Although scSemiGAN roughly divides various cell types into different regions, the compactness of each cell type is insufficient, with clear boundaries persisting between data from different batches. In contrast, scSemiPLC aggregates the data from both batches into a unified entity, although not fully merged, reducing the impact of platform inconsistency. Meanwhile, all cells are correctly divided into six distinct clusters, matching their respective cell types. This experiment demonstrates that scSemiPLC can produce robust representations to mitigate the technical platform interference. We will introduce additional modules to enhance the model’s integration ability regarding irrelevant factors in the data.

### Effect of the percent of labeled cells

In practical scenarios, obtaining a large amount of labels that satisfy the criteria leads to high costs. Therefore, achieving stable and high-quality annotation performance with only a small amount of labeled data is a common pursuit among different cell annotation methods.

**Fig 5:**
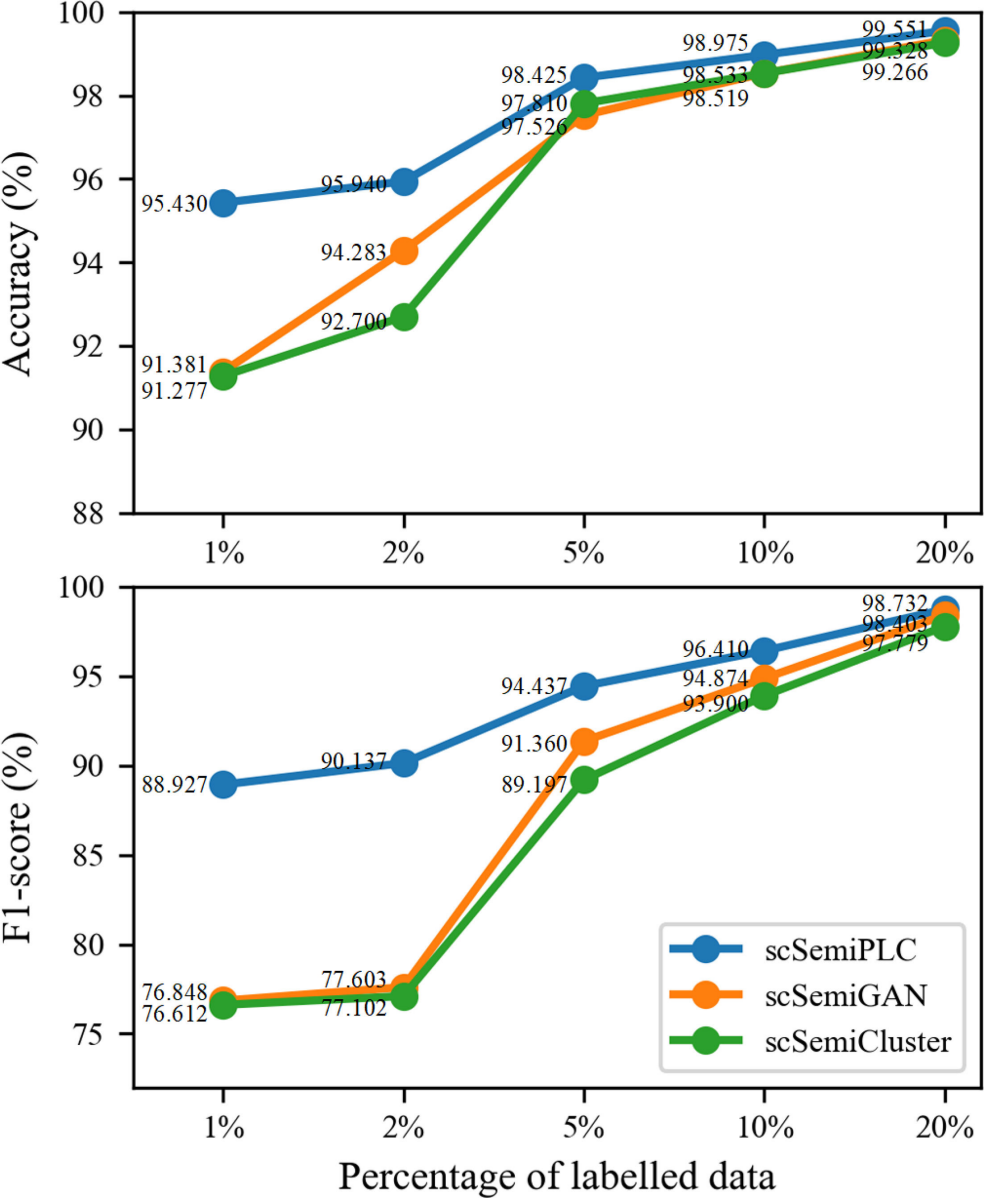
Annotation performance of scSemiPLC, scSemiGAN, and scSemiCluster with different percentages of labeled cells.

We use p to denote the proportion of labeled cells in the total number of samples. Here, we present the experimental results on the Kidney dataset. We set p = 0.01, 0.02, 0.05, 0.1, and 0.2 to select cell labels randomly. For each value of p, we repeat the experiment 10 times and calculate the average accuracy of cell annotation, as shown in Fig. 5. The annotation performance curves of scSemiGAN and scSemiCluster decline significantly when p<0.05, while scSemiPLC remains relatively flat, indicating that scSemiPLC exhibits better robustness to the number of cell labels. The accuracy and F1 score of scSemiPLC become stable when p>0.05, suggesting that it is capable of achieving satisfactory annotation performance with only a small amount of labeled data. Additionally, we record the average runtime for each execution of these methods. For the Kidney dataset with 2,781 cells, scSemiGAN and scSemiCluster take 4.54 and 6.76 min, respectively, while scSemiPLC completes in just 2.18 min on the same machine, demonstrating the efficiency advantage of scSemiPLC.

### Ablation study and analysis

To explore the importance of (1) contrastive learning-based pretraining, (2) consistency regularization, and (3) confidence evaluation in model training, we conduct an ablation study on scSemiPLC to demonstrate the impact of missing different parts on the cell annotation results. From the results in Table 3, with 10% of the data labels, the average F1-score 87.544 and accuracy 93.690 of removing consistency regularization over eight datasets are lower than the average F1-score 92.031 and accuracy 94.624 of the scSemiPLC framework. For 1 and 3, we observe the experimental results with the default 10% data labeling and further investigate the impact on annotation performance as the proportion of labeled data decreases. For the impacts caused by the absence of 1 and 3, we explore the variation in model annotation performance with different numbers of cell labels. Taking the Kidney and Baron Mouse datasets as examples, the results in Table 4 and Table S2 illustrate that as the number of cell labels decreases, the annotation performance declines due to the absence of 1 and 3. When the proportion of cell labels in the Kidney dataset decreases from 0.1 to 0.01, the presence of 1 reduces the decline in the F1-score of scSemiPLC by 2%, and the presence of 3 reduces the decrease in accuracy by 0.8%. These results consistently demonstrate the role of pretraining and confidence evaluation in enhancing model stability and annotation performance, especially for sparse cell labels.

**TABLE 3:** Ablation study of the II component on different data sets with 10% labels

Data set		Baron Mouse	Bladder	PBMC_10x	Kidney	PBMC SeqWell	Tongue	Baron Human	Chen
w/o II	Acc	94.411	99.668	80.330	98.946	89.377	98.343	98.031	90.416
	F1	81.173	98.569	76.081	95.374	77.891	98.132	87.197	85.935
scSemiPLC	Acc	96.909	99.900	83.548	98.975	91.061	98.346	98.363	89.893
	F1	91.101	99.760	84.968	96.410	85.001	98.725	93.039	87.242

**TABLE 4:** Effect of self-supervised pretraining and confidence assessment on annotation performance at different numbers of cell labels (Kidney)

Percentage		0.01	0.02	0.05	0.1
w/o I	Acc	94.286 (−1.147)	95.282 (−0.658)	98.012 (−0.413)	98.889 (−0.086)
	F1	86.798 (−2.129)	88.378 (−1.759)	93.673 (−0.764)	96.258 (−0.152)
w/o III	Acc	94.642 (−0.788)	95.872 (−0.068)	98.324 (−0.101)	98.885 (−0.09)
	F1	88.065 (−0.862)	89.824 (−0.313)	94.161 (−0.276)	96.195 (−0.215)
scSemiPLC	Acc	95.43	95.94	98.425	98.975
	F1	88.927	90.137	94.437	96.41

In summary, removing any part degrades the performance of cell annotation, particularly for consistency regularization. Such a result suits the practical situation, as consistency regularization is essential for performing semi-supervised annotation using unlabeled data. Removing this component means that the unlabeled data are only trained as a whole during pretraining, without precisely fine-tuning the acquired knowledge. The three components are complementary during training and can be used in combination to obtain cell annotation results with higher confidence.

### Conclusion

Researchers can explore more details about cell composition because of the higher resolution of gene expression at the cell level. As a crucial step in scRNA-seq data analysis, cell annotation is a prerequisite to support valuable biological discoveries. However, traditional manual annotation is time-consuming and cannot cope with the challenges caused by the increasing volume of data. Therefore, it is essential to explore automatic cell annotation methods that can be applied to different datasets.

As a semi-supervised training framework based on clustering to produce pseudo-labels, scSemiPLC makes full use of massive unlabeled data to learn the intrinsic regularities and representation of the data for better cell annotation. To utilize the unlabeled data, scSemiPLC employs a pseudo-labeling paradigm to assign labels to the unlabeled data, enabling it to participate in model training through supervised learning. Unlike previous semi-supervised methods, scSemiPLC calculates initial cluster centers from labeled data, obtains pseudo-labels through iterative clustering, and evaluates these pseudo-labels through a confidence module. This approach considers both the quantity and quality of pseudo-labels.

Experimental results on eight real scRNA-seq datasets demonstrate the more substantial competitiveness and superior performance of scSemiPLC compared to other benchmark methods. It consistently achieves the highest accuracy and F1-score in cell annotation and further amplifies these advantages when the labeled data are reduced. Additionally, visualizations of the extracted representations demonstrate that scSemiPLC can learn biologically relevant cell-type embeddings, exhibiting superior feature extraction performance compared to other methods. With respect to the interference from non-concerned variables, such as technology platforms, scSemiPLC demonstrates strong robustness. Despite these advantages, we will continuously optimize scSemiPLC to enhance its generalization capabilities further. Specifically, we attempt to introduce domain adaptation methods to address biases across heterogeneous data sources. Additionally, we will explore hierarchical learning mechanisms to improve robustness in complex scenarios with increased cell type diversity and larger sample sizes.

## Data Availability

All data for experiments in this study are publicly available scRNA-seq datasets. The Baron Mouse and Baron Human datasets are from GSE84133. The Bladder, Kidney, and Tongue datasets are from GSE109774. The PBMC_10x, PBMC_SeqWell, and Chen datasets are from the 10X Genomics website, GSE92495, and GSE87544, respectively. The scSemiPLC code is accessible and downloaded from https://github.com/WangDaMiao97/scSemiPLC.

## SUPPLEMENTAL MATERIAL

Supplemental File msystems.00223-25-s0001.docx Supplemental tables and figure.

ASM does not own the copyrights to Supplemental Material that may be linked to, or accessed through, an article. The authors have granted ASM a non-exclusive, world-wide license to publish the Supplemental Material files. Please contact the corresponding author directly for reuse.
