# Supplementary material for: scSemiPLC: a semi-supervised learning framework for annotating single-cell RNA-Seq data by generating pseudo-labels through clustering
Source: mSystems. 2025 Dec 8;11(1):e00223-25. doi: 10.1128/msystems.00223-25 (PMC12817951; doi:10.1128/msystems.00223-25)

Supplementary Table 1. Comparison of model performance across datasets

| Dataset | Baron Mouse | Bladder | PBMC10× | Kidney | PBMC SeqWell | Tongue | Baron Human | Chen |
| --- | --- | --- | --- | --- | --- | --- | --- | --- |
| scmap-cluster | 73.230 ** ±1.126 | 73.870 ** ±0.733 | 72.383 ** ±0.946 | 79.201 ** ±0.306 | 22.985 ** ±0.920 | 71.014 ** ±0.086 | 69.230 ** ±0.991 | 69.681 ** ±0.531 |
| scmap-cell | 44.327 ** ±0.800 | 67.938 ** ±2.310 | 58.251 ** ±0.497 | 64.898 ** ±0.865 | 45.607 ** ±0.891 | 48.995 ** ±0.136 | 60.373 ** ±0.453 | 62.448 ** ±0.601 |
| SingleR | 73.789 ** ±1.143 | 98.571 ** ±0.185 | 71.703 ** ±0.770 | 90.578 ** ±0.532 | 75.028 ** ±0.433 | 67.081 ** ±0.499 | 79.007 ** ±0.757 | 75.782 ** ±0.480 |
| SingleCellNet | 61.656 ** ±1.292 | 87.574 ** ±1.105 | 70.808 ** ±0.705 | 83.358 ** ±0.814 | 78.910 ** ±0.230 | 67.504 ** ±1.499 | 69.538 ** ±0.838 | 68.310 ** ±0.230 |
| scSemiCluster | 80.059 ** ±1.457 | 98.508 ** ±0.318 | 71.259 ** ±1.425 | 93.900 * ±0.814 | OCMM | 97.475 ** ±0.328 | 88.232 ** ±1.251 | 83.744 ** ±0.580 |
| CALLR | 77.433 ** ±1.417 | 96.625 ** ±0.627 | 61.881 ** ±4.399 | 94.741 ±1.027 | 79.995 ** ±0.417 | 97.161 ** ±0.140 | 70.506 ** ±1.076 | 78.619 ** ±0.019 |
| scSemiGAN | 66.219 ** ±1.864 | 97.607 ** ±0.731 | 68.449 ** ±0.978 | 94.874 ±0.619 | 76.391 ** ±0.978 | 97.136 ** ±0.485 | 83.674 ** ±1.078 | 86.452 ±0.609 |
| scSemiPLC | **91.101_±1.752_** | **99.760_±0.052_** | **84.968_±0.381_** | **96.410_±0.845_** | **85.001_±0.722_** | **98.725_±0.179_** | **93.039_±0.984_** | **87.242_±0.679_** |

The data in the table show the average F1-score (±standard error) of the ten-fold cross-validation. Bold text marks the best models per task. OCMM indicates that the original count matrix required by scSemiCluster is missing from the PBMC_SeqWell dataset. The t-test is conducted to compare the performance of each method against scSemiPLC. Statistical significance is indicated with ** for p<0.01 and * for p<0.05.

**Supplementary Table 2.** Effect of self-supervised pre-training and confidence assessment on semi-supervised learning at different numbers of cell labels(Baron Mouse)

|  | | 0.01 | 0.02 | 0.05 | 0.1 |
| --- | --- | --- | --- | --- | --- |
| w/o Ⅰ | Acc | 92.656(-1.066) | 94.003(-0.408) | 94.719(-0.673) | 96.734(-0.175) |
|  | F1 | 84.924(-2.206) | 85.000(-2.932) | 85.699(-2.514) | 90.619(-0.482) |
| w/o Ⅲ | Acc | 93.462(-0.260) | 94.274(-0.137) | 95.302(-0.09) | 96.898(-0.011) |
|  | F1 | 86.919(-0.211) | 88.034(+0.102) | 87.732(-0.481) | 91.092(-0.009) |
| scSemi  PLC | Acc | 93.722 | 94.411 | 95.392 | 96.909 |
|  | F1 | 87.130 | 87.932 | 88.213 | 91.101 |

**Supplementary Figure 1.** The internal architecture diagram of the confidence estimation module.


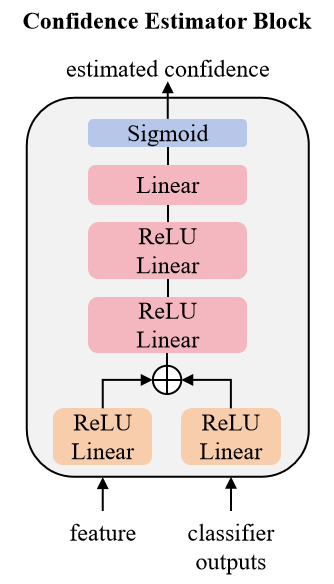

Supplement: Supplemental File — Supplemental tables and figure. [file msystems.00223-25-s0001.docx]
